# Optimized Measurement Parameters of Sensory Evoked Cortical Potentials to Assess Human Bladder Afferents - A Randomized Study

**DOI:** 10.1038/s41598-019-54614-z

**Published:** 2019-12-20

**Authors:** Stéphanie van der Lely, Martina D. Liechti, Melanie R. Schmidhalter, Martin Schubert, Lucas M. Bachmann, Thomas M. Kessler, Ulrich Mehnert

**Affiliations:** 1 Department of Neuro-Urology, Balgrist University Hospital, University of Zürich, Zürich, Switzerland; 2 Neurophysiology, Spinal Cord Injury Center, Balgrist University Hospital, University of Zürich, Zürich, Switzerland; 3 Medignition Inc., Research Consultants, Zürich, Switzerland

**Keywords:** Cortex, Bladder

## Abstract

Overactive bladder and voiding dysfunction are highly prevalent and often associated with malfunction of the bladder afferent pathways. Appropriate diagnostic tools for an objective assessment of afferent nerve function of the human bladder are currently missing. One promising possibility is the assessment of sensory evoked potentials (SEP) during repetitive electrical bladder stimulation, which proved feasible in healthy subjects. For an implementation into clinical practice, however, further refinements for efficient and reliable data acquisition are crucial. The aim of this randomized study was to find the optimal measurement settings regarding stimulation frequency, repetition number, and data acquisition. Forty healthy subjects underwent two visits of SEP (Cz-Fz) assessments using repetitive (500 stimuli) electrical stimulation of 0.5 Hz, 1.1 Hz, and 1.6 Hz and pulse width of 1 ms at the bladder dome or trigone. SEP analyses revealed higher amplitudes and better signal-to-noise ratio (SNR) with lower stimulation frequencies, while latencies remained unchanged. Decreasing amplitudes and SNR were observed with continuing stimulation accompanied by decreasing responder rate (RR). When applying stimuli at a frequency of 0.5 Hz, averaging across 200 stimuli revealed optimal reliability with best SNR, RR and sufficiently high amplitudes. This constitutes an optimal compromise between the duration of the assessment and SEP peak-to-peak amplitudes.

## Introduction

There is accumulating evidence that dysfunction of the bladder sensory nerves has a primary role in pathological conditions such as overactive bladder (OAB)^1–3^ that is characterized by urgency, urinary frequency, nocturia and urgency incontinence^4^. Due to the lack of an established, objective and reliable clinical assessment tool, it is currently difficult to detect alterations of afferent human bladder pathways. Consequently, the pathologic mechanisms behind OAB frequently remain unknown. Methods used so far for the investigation of sensory innervation, such as current perception threshold (CPT) assessment and urodynamic investigation, largely rely on subjective or semi-objective criteria^5,6^. A more objective and qualitative assessment tool of afferent nerve function are sensory evoked potentials (SEP)^7^. While feasibility of bladder SEP recordings was already demonstrated in healthy subjects^8–13^ and patients^14,15^, previous studies of our group reported that bladder stimulation at a relatively slow frequency of 0.5 Hz/pulse width = 1 ms, led to more reproducible cortical SEPs in female and male healthy subjects in contrast to a faster frequency of 3 Hz/pulse width = 0.2 ms^8,11^. This could be explained by the fact that bladder afferents comprise only myelinated Aδ-fibers and unmyelinated C-fibers, while faster conducting fibers are missing^16,17^. Nevertheless, it is difficult to draw a conclusion about the frequency effect in view of the different pulse widths requiring further studies. To ensure SEP reproducibility, averages of at least 500 stimuli and a repetition of at least two runs have been recommended for measurements of the upper and lower limbs^7^. As peripheral nerve stimulation can be applied with rates as high as 3 Hz due to their fast conducting nature, the time required to obtain an average of 500 stimulations amounts to a few minutes and is thus not critical in terms of keeping stimulation conditions constant.

Considering the indirect control of electrode placement via intravesical catheters, potential changes of bladder volume with time and the longer stimulation durations (up to six-fold increase) due to lower stimulation frequencies, bladder SEP measurements are more challenging. This study was performed in order to refine the methodology and to optimize the settings such as frequency and repetition number of stimulation in order to achieve a faster and reliable evaluation of viscero-sensory afferent bladder pathways. This is important for an implementation into clinical diagnostics and to minimize measurement bias through changes that occur over time. We aimed to evaluate the impact of different stimulation frequencies below 3 Hz at a constant pulse width of 1 ms, number of stimuli/runs and low-pass filters (200 Hz versus 70 Hz as previously used in our publications^8,11^) on SEPs from the bladder. We hypothesized that SEP waveform, responder rate (RR), reliability and signal-to-noise ratio (SNR) can be improved with optimized measurement parameters. Post-processing using a 70 Hz versus 200 Hz low-pass filter was expected to lead to smoother but corresponding SEP waveforms despite inessentially decreased amplitudes and increased latencies. The SEP findings from the bladder dome (BD) and trigone (TG) were compared to pudendal SEPs as a clinical reference standard.

## Material and Methods

This study was approved by the local ethics committee (Kantonale Ethikkommission Zürich; KEK-ZH-Nr. 2013-0518), registered at clinicaltrials.gov (Identifier: NCT02272309, date of registration: 22/10/2014), and performed in accordance with the Declaration of Helsinki. Study data were collected and managed using REDCap (Research Electronic Data Capture) electronic data capture tools^18^. All subjects provided written informed consent prior to inclusion.

### Subjects

Healthy volunteers were recruited through postings and advertisements on internet platforms and were invited for the screening visit to the Neuro-Urology, Spinal Cord Injury Centre at Balgrist University Hospital between October 2015 and June 2017. Study subjects had to be between 18 and 40 years old as well as in good mental and physical condition. This was defined as the absence of any lower urinary tract (LUT) symptom according to International Continence Society (ICS) terminology^4^, any urological or neurological diseases, urinary tract infection (UTI), previous surgery for urological or neurological reasons, and any regular medication intake (except contraceptive). The screening of the subjects included a complete medical history, vital signs, physical examinations (including examination of urogenital sensation, bulbocavernosus reflex, anal reflex, anal sphincter tone, and anal squeeze response), free uroflowmetry, post-void residual, Montreal-Cognitive-Assessment (MoCA), Hospital Anxiety and Depression Scale (HADS), International Prostate Symptom Score (IPSS) and a 3-day bladder diary (BLD)^19^. All included subjects fulfilled predefined cut-offs (Table 1).

**Table 1:** Baseline characteristics (n = 40, 20 females).

Baseline characteristics					Group differences		
	Women Dome	Men Dome	Women Trigone	Men Trigone	Gender-Dome	Gender-Trigone	Location
	(n = 10)	(n = 10)	(n = 10)	(n = 10)	p-value	p-value	p-value
*Age [years]*	22.7 (18.8–26.9)	22.1 (19.1–31.9)	23.7 (18.3–35.8)	25.2 (18.3–32.2)	0.579	0.280	0.181
*Height [m]*	1.7 (1.6–1.8)	1.8 (1.7–1.9)	1.7 (1.6–1.9)	1.7 (1.6–2.0)	0.025*	0.103	0.233
*Weight [kg]*	65.0 ± 4.3	73.5 ± 10.4	63.0 ± 13.0	71.3 ± 12.0	0.034*	0.155	0.552
***3-day bladder diary***							
Micturition frequency per 24 hours	6.4 ± 2.3	5.0 ± 1.6	6.1 ± 1.7	5.9 ± 2.8	0.124	0.823	0.696
Micturition volume per micturition [mL]	312 (238–550)	349 (246–517)	376 (178–487)	303 (209–1057)	0.481	0.393	0.989
Fluid intake per 24 hours [mL]	2140 (1373–4287)	2048 (783–4183)	2192 (1067–3283)	1887 (1567–7953)	0.571	0.631	0.766
***Questionnaires***							
*ICIQ-FLUTS/MLUTS+*							
Filling symptoms	1.5 (0–4)	—	1 (0–3)	—	—	—	1
Voiding symptoms	0 (0–2)	1 (0–4)	0 (0–3)	2 (0–6)	—	—	1/0.350
Incontinence symptoms	0 (0–2)	1 (0–4)	0 (0–2)	1 (0–3)	—	—	0.626/0.905
*IPSS*	—	1 (0–3)	—	1.5 (0–6)	—	—	0.693
*OAB-q SF*							
Symptoms	6 (6–7)	6 (6–15)	8 (6–10)	6 (6–16)	0.764	0.209	0.063
QoL	13 (13–14)	13 (13–17)	13.5 (13–17)	13 (13–18)	0.256	0.438	0.197
*HADS*							
Anxiety	2.5 ± 2.2	2.6 ± 1.2	4 ± 1.8	3.3 ± 2.0	0.902	0.418	0.061
Depression	0 (0–4)	2 (0–5)	1.5 (0–6)	0.5 (0–4)	0.106	0.529	1
*MoCA*	28.6 ± 1.3	28.5 ± 1.2	28.1 ± 1.0	28.8 ± 1.1	0.857	0.160	0.784
***Neuro-Urological examination***							
Urogenital sensation (intact/impaired)	10/0	10/0	10/0	10/0			
Bulbocavernosus reflex (intact/impaired)	10/0	10/0	10/0	9/1			
Anal reflex (intact/impaired)	10/0	10/0	10/0	10/0			
Anal sphincter tone (intact/impaired)	10/0	10/0	10/0	10/0			
Anal squeeze response (intact/impaired)	10/0	10/0	10/0	10/0			
***Free uroflowmetry***							
Voided volume [mL]	360 (171–1243)	350 (95–658)	465 (207–1195)	429 (170–1195)	0.739	0.734	0.561
Maximum flow rate [mL/s]	53.6 (27.4–62.9)	33.9 (11.1–77.4)	43.7 (14.5–79.4)	29.7 (22.3–34.3)	0.190	0.008*	0.244
Post void residual [mL]	5.4 (0–38.1)	2.2 (0–34)	1.5 (0–64.5)	12.5 (0–102.7)	0.938	0.532	0.511

All subjects fulfilled predefined cut-offs for study inclusion: MoCA score ≥ 26, HADS ≤ 7 each, IPSS ≤ 7, Bladder diary (BLD): $\frac{24h\,urinary\,frequency}{drinking\,volume\,[mL]\,}\le 0.0045$ with a maximum of 1x nocturia, mean volume per void >150 mL and absence of urinary incontinence or urgency. ^+^ due to different scoring systems, female and male subjects have not been compared. ICIQ = International Consultation on Incontinence Modular Questionnaire, FLUTS = Female lower urinary tract symptoms, MLUTS = Male lower urinary tract symptoms, IPSS = International Prostate Symptom Score, OAB-q SF = The Overactive Bladder Questionnaire short-form, QoL = Quality of life, HADS = Hospital Anxiety and Depression Scale, MoCA = Montreal Cognitive Assessment. Data are represented as mean ± standard deviation (SD) or median (range) where appropriate. Significant differences are marked: *p < 0.05.

In addition, standardized urological questionnaires including the International Consultation on Incontinence Modular Questionnaire modules (ICIQ-FLUTS, ICIQ-MLUTS) and the Overactive Bladder Questionnaire short-form (Swiss German OAB) were completed^19^.

### Study design

As shown in the protocol paper of the SENSORYII project comprising 5 LUT stimulation sites^19^, a power analysis yielded a total inclusion number of 10 subjects per stimulation site and gender group. Following study inclusion, the volunteers were invited for two identical visits (interval of 29.4 ± 8.5 days) whereby the daytime was held constant for the measurement visits (0 – 3 h). Subjects were randomly assigned to one of the five groups receiving stimulation either at one of the bladder (BD, TG) or urethral (proximal, membranous - males only, distal) stimulation sites (allocation ratio: 1:1:1:1:1, Supplementary Figure S1). In view of the clinical need for afferent nerve function assessments of the bladder, we here focus on the bladder SEPs. At the beginning of each visit, UTI and pregnancy were excluded. All subjects were told to refrain from the consumption of caffeine and cigarettes three hours prior to the measurement and alcohol one day prior to the measurement. A follow-up interview was conducted after each measurement visit to assess wellbeing and potential adverse events of the volunteers. The last follow-up was performed in August 2017.

### Electrical stimulation

A custom-made 14 French stimulation catheter (Unisensor AG, Attikon, Switzerland) was inserted transurethrally into the bladder using non-anaesthetic lubricant gel. The catheter included radiopaque platinum electrodes to precisely position the catheter under fluoroscopic guidance^19^. By use of the catheter, the bladder was filled with 60 mL of contrast medium (Ultravist® 150TM, Bayer AG, Switzerland) at the beginning of each bladder SEP measurement to ensure constant starting volumes. During the assessments, subjects were lying quietly in the supine position with eyes closed. Constant current stimulation was generated using a neurophysiological stimulator (Dantec Keypoint Focus, Neurolite AG, Belp, Switzerland) and applied via the stimulation catheter. Repetitive square wave stimuli of different stimulation frequencies (0.5 Hz, 1.1 Hz, 1.6 Hz; pulse width = 1 ms) were applied at the predefined stimulation site. Following a repeated-measures, randomized controlled factorial design, the order of the stimulation frequencies was pseudorandomly allocated using a computer-generated randomization list stratified on gender. The research team performed sequence generation and randomisation and was not formally blinded to group allocation. Five consecutive runs of 100 stimuli each (run1–run5) were applied per frequency. This resulted in stimulation cycle durations of 16.7 min, 7.6 min, and 5.2 min for 0.5 Hz, 1.1 Hz, and 1.6 Hz, respectively. To ensure a stable catheter position during measurements, the catheter was taped to the upper leg and/or penis. At the beginning of each assessment, CPTs were determined at least two times as described previously^20^. After a one-off pain threshold (PT) assessment, stimulation intensities were individually adjusted aiming at a strong but non-painful stimulation. At the end of each stimulation, the bladder was emptied. Following bladder stimulations, three measurements of somatosensory evoked potentials (SSEPs) were performed at a frequency of 3.1 Hz in a random order: tibial nerve (pulse width = 0.2 ms); pudendal nerve (pulse width = 0.2 ms); pudendal nerve (pulse width = 1.0 ms). Similar to bladder SEP measurements, five runs of 100 stimuli were applied (duration: 2.7 min) per stimulation cycle. For our analysis, pudendal SEPs with 1 ms pulse width were selected as comparator to bladder SEPs due to the same pulse width.

### Recording

A surface electrode system comprising of a cap-based extended international 10–20 montage^21^ (Easy Cap, Easy Cap GmbH, Herrsching, Germany) was used. Electrooculogram and Electrocardiogram were also recorded. Continuous recordings of SEPs were performed from surface electrodes (Ag/AgCl) at Cz referenced to Fz using BrainVision Recorder (BrainProducts, Gilching, Germany). The ground electrode was placed at AFz position. Electrode impedances were kept below 20 kΩ. Amplification of the scalp electrode signals was performed using BrainAmp amplifier (Brain Products, Gilching, Germany). Sampling frequency was 5000 Hz and the applied analogue filter between 0.016 and 1000 Hz.

### Data processing and analyses

BrainVision Analyzer2 (BrainProducts, Gilching, Germany) was used for SEP processing. **Filtering** involved the application of 0.5 Hz-70 Hz band-pass (infinite impulse response filters; Butterworth zero-phase shift filter; 24 dB/Oct) plus 50 Hz Notch filter. This was followed by **ocular correction**^22^ and semiautomatic **artefact rejection** ( ± 100 µV) including visual inspection. To assure that no relevant bladder SEP component was lost, explorative analyses were performed using a 200 Hz instead of 70 Hz low-pass filter.

#### Segmentation and Averaging

Bladder SEP recordings were divided into 700 ms segments (100 ms pre-stimulus, 600 ms post-stimulus), while SSEP recordings were broken down into 300 ms segments (100 ms pre-stimulus, 200 ms post-stimulus). The segments of each individual run and of concatenated runs (run1 to run2 (run1_2), run1 to run3 (run1_3), run1 to run4 (run1_4), (run1 to run5 (run1_5)) were averaged and analyzed. We aimed for SEP averages consisting of at least 70% artefact free segments for each stimulation frequency, individual run and visit. For the direct filter comparison (i.e. 200 Hz vs. 70 Hz low-pass), only bladder SEP and pudendal SEP data sets retaining 100% of the segments after raw data inspection in both filter variants were included. Additionally, the averages of odd and even segments were evaluated for each run.

In order to exclude effects of different experimental durations, separate analyses were conducted on the averaged segments of the first 300 seconds of each stimulation cycle in order to directly compare bladder stimulation frequencies, independent from different stimulation durations. Exploratory analyses were performed using baseline correction (pre-stimulus time interval -53 ms to -3 ms) prior to averaging.

#### Peak detection and outcome measures

An SEP was regarded ‘stable’ if the averaged SEP signal of the odd and even segments overlapped in shape and timing with respect to the SEP components. **Peak detection** was performed manually. The markers were set using the following criteria: a) overlapping waveform between odd and even SEP averages and b) identifiable P1, N1, P2 bladder SEP components, respectively P40, N50, P65, N85 SSEP components. On a subject level, we were careful to choose individually corresponding SEP components across runs, frequencies and visits. Markers were individually set for the two filter versions on every single run and the combined runs. Subsequently, P1, N1, and P2, respectively P40, N50, P65 and N85 **peak latencies** and **peak-to-peak amplitudes** (P1N1 and P2N1 respectively P40N50 and P65N85) were extracted. **Responder rate** reflects the percentage of recordings that resulted in a stable SEP with identifiable components and clear marker placement. **Signal-to-noise ratio** of the Cz-Fz channel was calculated from the quotient of the average signal power and the average noise power for all locations, visits, frequencies, and runs, respectively. **Relative stimulation intensity** was calculated by dividing absolute stimulation intensity by CPT. **Produced volume** represents the emptied volume after a stimulation cycle minus the starting volume of 60 mL.

### Statistics

Preprocessing and statistical analyses were performed using BrainVision Analyzer 2 (BrainProducts, Gilching, Germany), RStudio (Version 1.1.453, Boston, MA, U.S.A.) and the Stata statistics software package 14.2 (StataCorp. 2015. Stata Statistical Software: Release 14. College Station, TX: StataCorp LP.). Continuous variables are presented with means and standard deviations (SD) (or median and range where appropriate). Normal distribution was tested using Shapiro-Wilk test and by visual inspection of histogram and qq-plots. For all statistical analyses, a significance level of p < 0.05 was used. Unpaired Welch’s t-test or Mann-Whitney-U tests were performed to check for gender and location differences. In order to compare baseline corrected SEP curves among the different stimulation frequencies, runs and visits including all subjects (even if marker setting was not possible), paired t-tests were calculated for each data point of the whole segment (t-curves). In graphical representations, significant t-values (two-tailed) are highlighted.

Wilcoxon signed rank tests were used to compare the two filter variants regarding SEP latency and amplitude measures. For SNR analysis, Friedman's test respectively Wilcoxon signed rank test (p-values Bonferroni corrected) were used to compare different frequencies, runs and visits.

#### Linear mixed modelling (LMM)

For the amplitude analysis, we considered the absolute peak-to-peak amplitudes for P1N1 and P2N1. For the latency analysis, those for P1, N1 and P2 were considered. We *a priori* defined the default settings of 0.5 Hz stimulation and the aggregation of 100 stimuli as comparator to the other frequencies and individual runs. We defined indicator variables for 1.1 Hz and 1.6 Hz, and eight indicator variates for other data aggregations (separate runs: second to fifth, and combined runs (run1_2), (run1_3), (run1_4) and (run1_5)). The following subjects’ characteristics were entered as additional independent variables: (age, female gender, body height, body weight, urine production volume and absolute stimulation intensity). Using this modelling set-up, we examined the effect of various measurement settings, data aggregations (runs) and subjects’ characteristics on amplitude and latency. We performed stratified analyses for visits (first vs. second), and for location (TG vs. BD). Analyses were run on complete cases only, excluding measurements with missing values. To account for repeated measurements between subjects, we introduced an indicator variate for study subject as a random factor to the model. Sensitivity analyses were performed by excluding one subject with extreme latency values and by introducing the relative instead of the absolute stimulation intensity to the model. LMMs of pudendal SEP data were performed similarly (without independent variable urine production volume) on complete cases. Due to the datamatrix, p-values should only be applied indicatively.

#### Reliability

Reliability across visits was analyzed using intraclass correlation coefficient (ICC, single measures, two-way random effect, and absolute agreement). The ICC values were characterized according to Cicchetti (1994)^23^ and consequently considered as “poor” (less than 0.40) “fair” (0.40 – 0.59), “good” (0.60 – 0.74) or “excellent” (0.75 – 1.00).

## Results

### Group descriptives

For the analysis of bladder SEPs, forty healthy controls were included (Supplementary Figure S1). Descriptive statistics are displayed in Table 1. All subjects tolerated the procedures well and no UTI was reported. However, 27 out of 40 subjects reported temporary self-limiting dysuria after 48 out of 80 measurements. Two subjects reported temporary self-limiting hematuria after 2 out of 80 measurements. All described symptoms were mild and expected due to irritations caused by the catheter, did not require medical consultation or treatment, and were continously declining over a short period of time, i.e. no more than 3 days.

Stimulation parameters and bladder volume are listed in Supplementary Table S1 stratified for gender, location and frequency.

### The impact of filter parameters

Typical and stable SEP components (P1, N1, P2 for bladder SEPs; P40, N50, P65, N85 for pudendal SEPs respectively) were detectable for both low-pass filter variants (Supplementary Figure S2). The 200 Hz and 70 Hz low-pass filter variant revealed similar SEP curve shapes along the entire SEP segments. Based on the marker positions, Wilcoxon-signed rank tests showed no filter-specific differences for the latencies of the bladder SEP and pudendal SEP components. Analysis of peak-to-peak amplitudes revealed no significant differences for bladder SEPs but smaller amplitudes for pudendal SEPs (P40N50: V = 0, p = 0.004; P65N85: V = 0, p = 0.004) preprocessed with 70 Hz compared to 200 Hz low-pass filter. Subjectively, manual marker setting was easier using 70 Hz low-pass filter due to the smoother SEP curve compared to the 200 Hz. Consequently, and considering the integrity of all relevant SEP components, the following results are presented for the 70 Hz low-pass filter variant.

### Number of segments

For the analyses of the effect of stimulation frequencies, number of runs, and visits, on the SEPs, the averages contained at least 70 % artefact free segments, except for five bladder SEP datasets which showed a lower number of valid segments in the first run (minimum of 46 %). Regarding pudendal SEPs, two datasets containing single runs with a minimum of 40 % of valid segments were included. Considering that the respective datasets showed stable SEPs, they were included in the main analysis.

### The impact of stimulation frequency on SEP outcome

**Figure 1:**
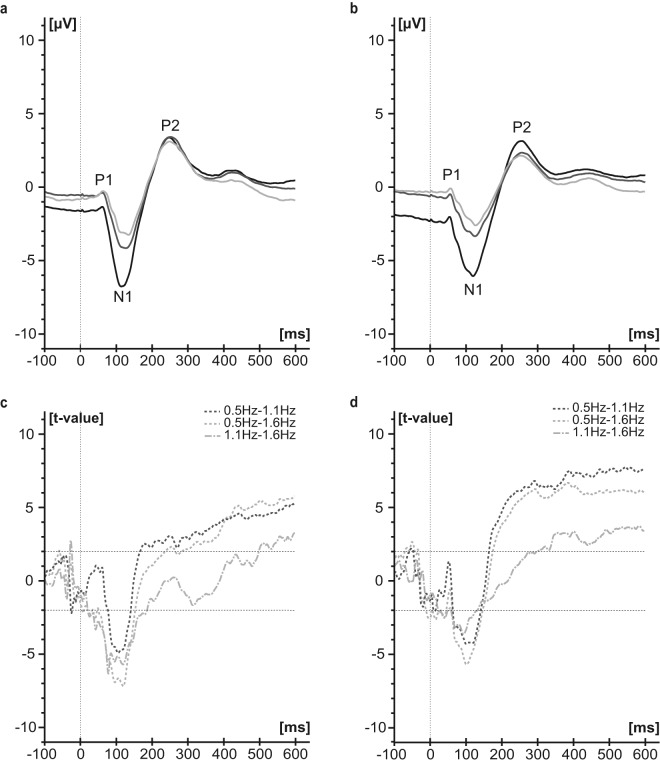
SEP group averages across two visits and a constant number of 500 stimuli. SEPs were recorded from Cz-Fz during stimulation at the BD (**a**, n = 20 subjects) and TG (**b**, n = 20 subjects) with the three different stimulation frequencies: 0.5 Hz (black), 1.1 Hz (dark grey) and 1.6 Hz (light grey, all without baseline correction). T-curves of differences between the stimulation frequencies (baseline corrected; 0.5 Hz-1.1 Hz: dark grey dashed line; 0.5 Hz-1.6 Hz: light grey dashed line; 1.1Hz-1.6 Hz: light grey dotted dashed line) are shown for BD (**c**) and TG (**d**). T-values above 2.021 and below -2.021 were considered as significant.

Typical P1, N1, P2 components were found with a 100 % RR for all three stimulation frequencies and both locations with larger peak-to-peak amplitudes when using slower stimulation frequencies (Fig. 1a,b).

T-curves of the SEP differences between the frequencies are illustrated in Fig. 1 for BD (c) and TG (d). Significant differences between the frequencies were prominent around the N1 and P2 peak. For the N1 peak we observed differences between all frequency comparisons. For the P2 peak, significant differences were observed only between 0.5 Hz and 1.1 Hz as well as 0.5 Hz and 1.6 Hz. Slow stimulation frequency consistently presented with a negative shift during the baseline phase (e.g. 0.5 Hz SEP curves in Fig. 1a,b).

Linear mixed model revealed smaller P2N1 peak-to-peak amplitudes during stimulation with 1.1 Hz (estimates between −3.5 µV and −4.5 µV, Tables 2a) and 1.6 Hz (estimates between -5.1 µV and -5.9 µV, Table 2a) compared to 0.5 Hz. Latencies of the N1 peak were similar between 0.5 Hz and 1.1 Hz as well as 0.5 Hz and 1.6 Hz (Table 2b).

**Table 2:** Results of the linear mixed effect models (LMM) showing the influence of different variables on P2N1 amplitude (a) and N1 latency (b).

a) P2N1 amplitude	Dome, 1.visit				Dome, 2. visit				Trigone, 1. visit				Trigone, 2. visit			
	Estimate [μV]	Pr( > |t|)	CI lower	CI Upper	Estimate [μV]	Pr( > |t|)	CI lower	CI Upper	Estimate [μV]	Pr( > |t|)	CI lower	CI Upper	Estimate [μV]	Pr( > |t|)	CI lower	CI Upper
(Intercept)	1.43	0.94	−40.77	43.63	33.99	0.27	−28.04	96.03	10.59	0.52	−22.91	44.09	26.55	0.21	−16.03	69.14
Freq. 1.1 Hz	−3.74	0.00*	−5.84	−1.63	−3.60	0.00*	−5.61	−1.59	−3.54	0.00*	−5.09	−1.99	−4.53	0.00*	−6.24	−2.82
Freq. 1.6 Hz	−5.87	0.00*	−9.14	−2.60	−5.87	0.00*	−8.47	−3.28	−5.09	0.00*	−7.68	−2.51	−5.57	0.00*	−8.33	−2.81
Run2	−1.82	0.00*	−2.65	−0.99	−1.72	0.00*	−2.62	−0.81	0.33	0.67	−1.25	1.90	−0.79	0.14	−1.86	0.28
Run3	−2.50	0.00*	−3.45	−1.54	−2.92	0.00*	−3.94	−1.89	−0.62	0.38	−2.08	0.83	−1.61	0.00*	−2.58	−0.64
Run4	−2.73	0.00*	−3.63	−1.82	−3.92	0.00*	−5.04	−2.80	−0.87	0.27	−2.47	0.73	−1.85	0.00*	−2.95	−0.75
Run5	−3.94	0.00*	−5.26	−2.61	−4.32	0.00*	−5.43	−3.21	−0.92	0.20	−2.37	0.53	−2.37	0.00*	−3.60	−1.14
Run1_2	−1.11	0.00*	−1.56	−0.67	−1.05	0.00*	−1.52	−0.59	−0.17	0.64	−0.93	0.59	−0.63	0.01*	−1.10	−0.17
Run1_3	−1.86	0.00*	−2.48	−1.23	−1.95	0.00*	−2.66	−1.24	−0.65	0.18	−1.64	0.33	−1.18	0.00*	−1.79	−0.56
Run1_4	−2.38	0.00*	−3.19	−1.58	−2.73	0.00*	−3.60	−1.86	−0.96	0.08	−2.04	0.12	−1.54	0.00*	−2.25	−0.83
Run1_5	−2.91	0.00*	−3.82	−2.01	−3.41	0.00*	−4.38	−2.44	−1.16	0.04*	−2.29	−0.03	−1.85	0.00*	−2.63	−1.08
Gender−male	−3.63	0.01*	−6.46	−0.79	−3.62	0.01*	−6.39	−0.86	2.38	0.19	−1.30	6.07	2.57	0.20	−1.44	6.58
Age	−0.62	0.00*	−0.94	−0.31	−0.77	0.01*	−1.28	−0.26	−0.20	0.20	−0.53	0.12	−0.05	0.72	−0.36	0.25
Body weight	0.13	0.29	−0.12	0.38	0.25	0.14	−0.09	0.58	0.08	0.51	−0.18	0.34	0.13	0.36	−0.17	0.43
Volume	−0.01	0.48	−0.03	0.01	−0.02	0.04*	−0.05	0.00	0.01	0.64	−0.02	0.03	0.00	0.70	−0.01	0.01
Body height	0.14	0.39	−0.19	0.46	−0.08	0.73	−0.54	0.39	−0.01	0.95	−0.28	0.26	−0.12	0.48	−0.47	0.23
Intensity	−0.11	0.12	−0.26	0.03	0.02	0.82	−0.19	0.24	−0.05	0.43	−0.19	0.08	−0.11	0.06	−0.22	0.01
Adjusted R^2^ = 0.415/0.399/0.326/0.307																
**b) N1 latency**	**Dome, 1.visit**				**Dome, 2. visit**				**Trigone, 1. visit**				**Trigone, 2. visit**			
	**Estimate [ms]**	**Pr(** > **|t|)**	**CI lower**	**CI Upper**	**Estimate [ms]**	**Pr(** > **|t|)**	**CI lower**	**CI Upper**	**Estimate [ms]**	**Pr(** > **|t|)**	**CI lower**	**CI Upper**	**Estimate [ms]**	**Pr(** > **|t|)**	**CI lower**	**CI Upper**
(Intercept)	78.82	0.14	−29.29	186.93	89.01	0.07	−9.76	187.80	85.47	0.01	29.04	141.89	96.55	0.05	−1.97	195.08
Freq. 1.1 Hz	4.16	0.05	0.01	8.31	1.92	0.48	−3.67	7.50	2.44	0.22	−1.57	6.44	3.29	0.03*	0.32	6.26
Freq. 1.6 Hz	5.43	0.10	−1.24	12.09	−0.41	0.88	−5.95	5.14	3.46	0.15	−1.39	8.31	1.87	0.40	−2.66	6.41
Run2	1.33	0.01*	0.38	2.29	0.53	0.43	−0.86	1.92	−1.00	0.28	−2.86	0.87	−0.53	0.42	−1.86	0.80
Run3	1.94	0.02*	0.28	3.60	0.54	0.28	−0.47	1.56	−0.94	0.13	−2.19	0.30	−0.58	0.48	−2.26	1.11
Run4	1.51	0.11	−0.35	3.38	0.49	0.53	−1.10	2.08	−0.69	0.50	−2.78	1.40	−1.31	0.19	−3.32	0.70
Run5	0.88	0.30	−0.86	2.61	1.10	0.21	−0.66	2.86	−0.70	0.41	−2.40	1.01	−2.36	0.04*	−4.60	−0.12
Run1_2	0.84	0.00*	0.32	1.35	0.58	0.22	−0.37	1.53	0.12	0.86	−1.29	1.53	−0.35	0.52	−1.48	0.78
Run1_3	0.63	0.02*	0.13	1.13	0.69	0.19	−0.37	1.75	0.17	0.80	−1.15	1.49	−0.19	0.73	−1.32	0.94
Run1_4	0.69	0.04*	0.05	1.34	0.79	0.16	−0.34	1.92	0.17	0.81	−1.27	1.60	−0.40	0.54	−1.72	0.93
Run1_5	0.91	0.01*	0.27	1.56	1.20	0.04*	0.05	2.34	0.22	0.75	−1.18	1.61	−0.21	0.75	−1.61	1.18
Gender−male	−6.69	0.34	−20.99	7.61	−10.66	0.06	−21.68	0.36	8.99	0.07	−0.65	18.63	8.74	0.18	−4.31	21.79
Age	0.80	0.28	−0.72	2.32	0.76	0.23	−0.52	2.04	−2.00	0.00*	−2.81	−1.18	−2.23	0.00*	−3.57	−0.90
Body weight	−0.15	0.74	−1.05	0.76	0.08	0.78	−0.50	0.65	−0.93	0.00*	−1.50	−0.37	−0.89	0.02*	−1.58	−0.19
Volume	0.03	0.28	−0.03	0.09	−0.03	0.09	−0.07	0.01	−0.02	0.39	−0.07	0.03	−0.01	0.52	−0.05	0.02
Body height	0.17	0.67	−0.66	1.01	0.10	0.78	−0.64	0.84	0.77	0.00*	0.35	1.20	0.74	0.03*	0.07	1.41
Intensity	−0.09	0.79	−0.81	0.63	−0.17	0.44	−0.64	0.29	0.54	0.02*	0.10	0.99	0.25	0.23	−0.17	0.68
Adjusted R^2^ = 0.194/0.292/0.606/0.523																

Significant differences are marked: *p < 0.05. Volume = Produced volume in mL, Intensity = absolute stimulation intensity in mA.

Results of the LMM for P1N1 amplitude as well as P1 and P2 latencies are shown in supplementary Table S2–S4. Median peak latencies for the location BD across visits and the three frequencies (500 stimuli) were 65.2 ms (47.0 to 84.2 ms) for P1, 117.5 ms (97.6 to 150.4 ms) for N1 and 250.2 ms (209.4 to 298.6 ms) for P2. Median latencies for the location TG were 57.5 ms (48.2 to 97.6 ms) for P1, 118.2 ms (94.6 to 178.4 ms) for N1 and 252.5 ms (216.2 to 304.2 ms) for P2. Similar LMM results were shown when excluding one subject with extreme values or when introducing the relative rather than the absolute stimulation intensity to the model.

**Figure 2:**
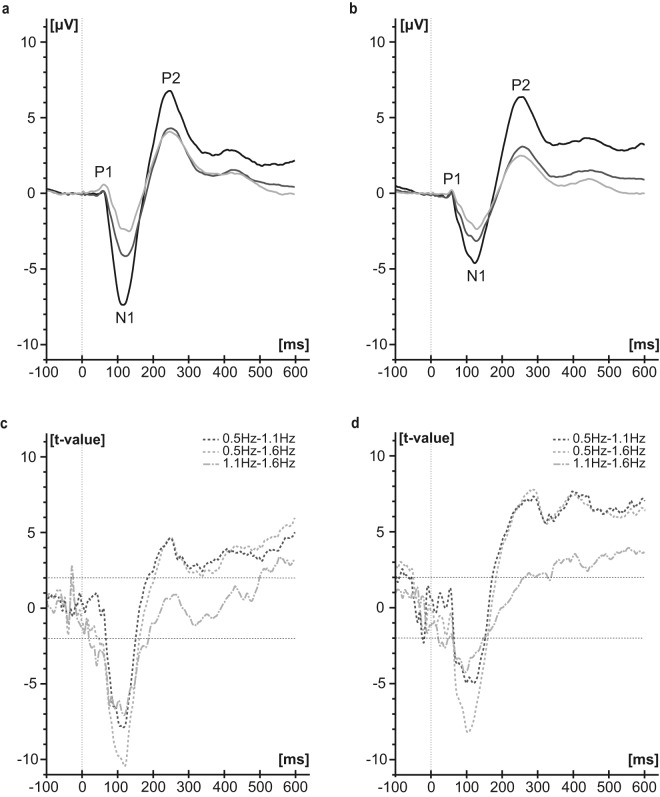
Baseline corrected SEP group averages across two visits after a constant stimulation duration of 300 seconds. SEPs were recorded from Cz-Fz during stimulation at the BD (**a**, n = 20 subjects) and TG (**b**, n = 20 subjects) with the three different stimulation frequencies: 0.5 Hz (black), 1.1 Hz (dark grey) and 1.6 Hz (light grey). T-curves of differences between the stimulation frequencies (baseline corrected, 0.5Hz-1.1 Hz: dark grey dashed line; 0.5Hz-1.6 Hz: light grey dashed line; 1.1Hz-1.6 Hz: light grey dotted dashed line) are shown for BD (**c**) and TG (**d**). T-values above 2.021 and below -2.021 were considered as significant.

Across both locations, SNR of the whole SEP curve (Cz-Fz, baseline corrected) of the first visit was significantly higher for lower frequencies (0.5 Hz: 0.14 (0.01–1.47); 1.1 Hz: 0.07 (0.01–0.59); 1.6 Hz: 0.04 (0.00–0.48); Friedman-test chi-squared: 31.65, df = 2, p < 0.001; 0.5Hz-1.1 Hz: V = 779, p < 0.001; 0.5Hz-1.6 Hz: V = 764, p < 0.001, 1.1Hz-1.6 Hz: V = 540, p = 0.25; Bonferroni corrected) compared to higher stimulation frequencies. Separate analyses over a fixed stimulation duration of 300 s showed similar frequency effects compared to a fixed number of stimuli as can be seen in Fig. 2.

### Impact of number of stimuli on SEP outcome

**Figure 3:**
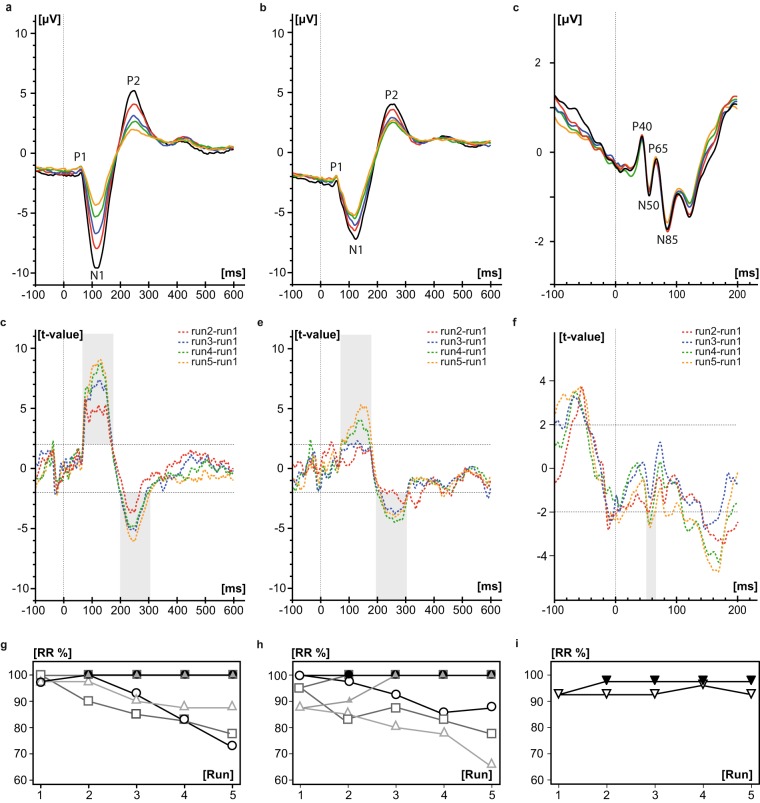
SEP group averages of the individual runs across two visits recorded from Cz-Fz during stimulation with 0.5 Hz at the BD (**a**, n = 20 subjects) respectively TG (**b**, n = 20 subjects) and 3.1 Hz at the pudendal nerve (**c**, n = 40 subjects). Run1, run2, run3, run4, and run5 are indicated in black, red, blue, green and orange, respectively (without baseline correction). T-curves of differences between baseline corrected individual runs 2–5 to run 1 (run2-run1: red dashed, run3-run1: blue dashed, run4-run1: green dashed, run5-run1: orange dashed) are shown for BD (**d**), TG (**e**) and pudendal nerve (**f**). T-values above 2.021 (for pudendus: 1.990) and below -2.021 (for pudendus: -1.990) were considered as significant. Significant differences between runs are highlighted in grey. Responder rate is displayed in % across the individual runs (open symbols) as well as the combined runs (filled symbols, consider that some symbols of the different frequencies overlay) of the runs for BD (**g**) and TG (**h**) and pudendal nerve (**i**) with 0.5 Hz indicated by black circles, 1.1 Hz, dark grey squares, 1.6 Hz, light grey up-pointing triangles, and 3.1 Hz, black down-pointing triangles, respectively.

Considering each of the five runs (each consisting of 100 stimuli) separately, decreasing amplitudes were observed from run 1 to 5 accompanied by decreasing RR for all locations and the three frequencies (Fig. 3). Sections with significant differences between runs are shown in Fig. 3d–f. While there were significant differences around the N1 and P2 peak between run1 and all other individual runs for the location BD, only the differences between run1, and run4 and run5, respectively, reached significance for the location TG. Pudendal SEPs showed significant reductions between run1 and run4, as well as run1 and run5, around 55 ms.

The analysis of the individual marker positions supported the findings of decreasing amplitudes from run1 to run5 (see estimates in Table 2a). For N1 latencies, estimates of differences were between -2.4 ms and 1.1 ms (Table 2b). For the pudendal nerve stimulation, LMM results are reported in Supplementary Table S5–S7.

Responder rate of bladder SEPs across runs is shown in Fig. 3g,h. By combination of the first two runs, RR can be maximized for all frequencies of the two locations except for the frequency 1.6 Hz during TG stimulation where the combination of three runs is necessary to reach a RR of 100%. These results were shown for both measurement visits. Responder rate of pudendal nerve stimulation could also be increased by combination of at least two runs; however, the highest RR reached was 97.5 % (Fig. 3i).

**Figure 4:**
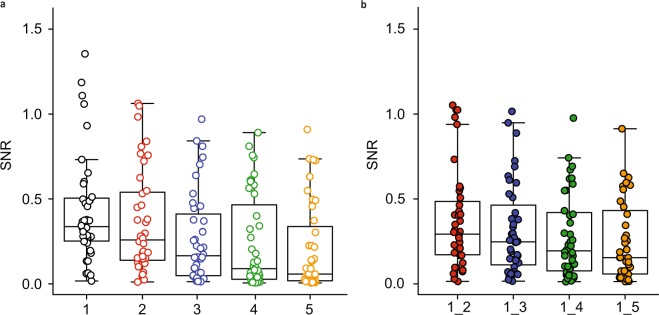
Signal-to-noise ratio (SNR) of baseline corrected data across two visits plotted for the individual (**a**) and combined runs (**b**; i.e. 1_2: averaged segments across run1 and run2) when stimulating at the BD with 0.5 Hz (n = 20 subjects).

The SNR of the entire SEP curve of the first visit was significantly different between runs across both bladder locations (Friedman chi-squared = 92.23, df = 4, p = <0.001) and for pudendal SEP measurements (Friedman chi-squared = 16.24, df = 4, p = 0.003) with earlier runs showing greater SNR. See Fig. 4 for SNR changes across runs for the location BD during stimulation with 0.5 Hz.

### Changes of SEP outcome measures across visits

**Figure 5:**
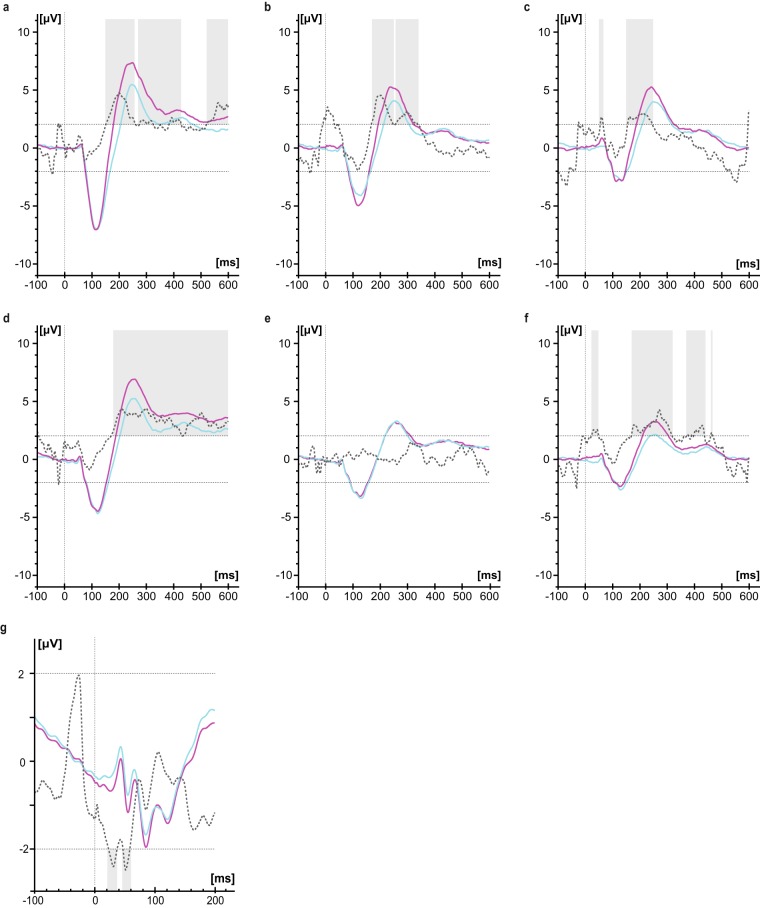
Baseline corrected SEP group averages of 200 stimuli recorded at Cz-Fz during 0.5 Hz, 1.1 Hz, and 1.6 Hz stimulation of the BD (**a**–**c**), TG (**d,e,f**) and pudendal nerve (**g**, exception: 500 stimuli). Visit one and two are indicated in light blue, respectively violet. The dashed grey lines constitute the corresponding t-values (visit2-visit1; baseline corrected). T-values above 2.086 (for pudendal SEP: 2.021) and below -2.086 (for pudendal SEP: -2.021) were considered as significant. Significant differences between visits are highlighted in grey.

Between the two visits, group averages showed good agreement of SEP waveform for the two bladder locations and the pudendal nerve (Fig. 5). While significant differences were reported for pudendal SEPs before P40 and between P40/N50, bladder SEPs showed significantly greater amplitudes during the second visit around the P2 peak, except for TG during 1.1 Hz stimulation.

When taking visit as an independent variable in the LMM, latencies of the second visit were slightly shorter for both locations (N1 - estimate BD: -2.0 ms, N1 - estimate TG: -1.1 ms, P40 – estimate pudendus: -0.3 ms) while amplitudes were greater (P2N1 - estimate BD: 1.3µV, P2N1 - estimate TG: 0.8 µV, P40N50 – estimate pudendus: 0.1 µV). Across all five runs, there was no significant difference in SNR between visits for all frequencies of the two bladder locations (p > 0.114) and the pudendal nerve (p = 0.685). The ICC values for latency and amplitude measures are given in Table 3 for bladder SEP and pudendal SEP measurements.

**Table 3:** Reliability of SEP components across visits.

Location	Marker	Run	0.5 Hz					1.1 Hz					1.6 Hz				
			n	ICC	Lower CI	Upper CI	ICC category	n	ICC	Lower CI	Upper CI	ICC category	n	ICC	Lower CI	Upper CI	ICC category
Dome	P1	1	19	0.588	0.185	0.820	fair	20	0.805	0.570	0.918	excellent	19	0.245	−0.132	0.595	poor
Dome	P1	1_2	20	0.705	0.389	0.872	good	20	0.623	0.252	0.833	good	20	0.322	−0.069	0.648	poor
Dome	P1	1_5	20	0.710	0.396	0.875	good	20	0.600	0.218	0.821	good	20	0.395	−0.008	0.699	poor
Trigone	P1	1	20	0.719	0.424	0.878	good	18	0.787	0.515	0.915	excellent	16	0.784	0.491	0.919	excellent
Trigone	P1	1_2	20	0.740	0.456	0.888	good	20	0.348	−0.114	0.681	poor	17	0.756	0.456	0.903	excellent
Trigone	P1	1_5	20	0.945	0.865	0.978	excellent	20	0.700	0.378	0.870	good	20	0.876	0.710	0.949	excellent
**Dome**	**N1**	**1**	**19**	**0.872**	**0.698**	**0.948**	**excellent**	**20**	**0.869**	**0.702**	**0.946**	**excellent**	**19**	**0.595**	**0.209**	**0.821**	**fair**
**Dome**	**N1**	**1_2**	**20**	**0.886**	**0.695**	**0.956**	**excellent**	**20**	**0.935**	**0.846**	**0.974**	**excellent**	**20**	**0.489**	**0.079**	**0.759**	**fair**
**Dome**	**N1**	**1_5**	**20**	**0.904**	**0.758**	**0.962**	**excellent**	**20**	**0.937**	**0.849**	**0.974**	**excellent**	**20**	**0.518**	**0.113**	**0.776**	**fair**
**Trigone**	**N1**	**1**	**20**	**0.898**	**0.763**	**0.958**	**excellent**	**18**	**0.883**	**0.715**	**0.955**	**excellent**	**16**	**0.718**	**0.357**	**0.892**	**good**
**Trigone**	**N1**	**1_2**	**20**	**0.904**	**0.778**	**0.961**	**excellent**	**20**	**0.853**	**0.665**	**0.939**	**excellent**	**17**	**0.869**	**0.665**	**0.951**	**excellent**
**Trigone**	**N1**	**1_5**	**20**	**0.912**	**0.792**	**0.964**	**excellent**	**20**	**0.817**	**0.592**	**0.924**	**excellent**	**20**	**0.951**	**0.833**	**0.982**	**excellent**
Dome	P2	1	19	0.751	0.462	0.896	excellent	20	0.773	0.470	0.907	excellent	19	0.495	0.090	0.765	fair
Dome	P2	1_2	20	0.780	0.531	0.906	excellent	20	0.748	0.454	0.893	good	20	0.469	0.074	0.745	fair
Dome	P2	1_5	20	0.858	0.677	0.941	excellent	20	0.772	0.515	0.902	excellent	20	0.494	0.102	0.759	fair
Trigone	P2	1	20	0.816	0.518	0.929	excellent	18	0.676	0.311	0.866	good	16	0.561	0.127	0.819	fair
Trigone	P2	1_2	20	0.878	0.712	0.951	excellent	20	0.700	0.382	0.870	good	17	0.619	0.231	0.841	good
Trigone	P2	1_5	20	0.851	0.668	0.938	excellent	20	0.775	0.515	0.905	excellent	20	0.780	0.531	0.906	excellent
Dome	P1N1	1	19	0.427	−0.037	0.735	fair	20	0.782	0.536	0.907	excellent	19	0.190	−0.285	0.587	poor
Dome	P1N1	1_2	20	0.554	0.148	0.798	fair	20	0.591	0.229	0.813	fair	20	0.219	−0.227	0.593	poor
Dome	P1N1	1_5	20	0.607	0.234	0.824	good	20	0.450	0.025	0.738	fair	20	0.111	−0.325	0.515	poor
Trigone	P1N1	1	20	0.774	0.519	0.904	excellent	18	0.429	−0.050	0.743	fair	16	0.750	0.420	0.905	excellent
Trigone	P1N1	1_2	20	0.831	0.621	0.929	excellent	20	0.537	0.124	0.789	fair	17	0.681	0.317	0.871	good
Trigone	P1N1	1_5	20	0.739	0.449	0.888	good	20	0.545	0.135	0.793	fair	20	0.548	0.153	0.793	fair
Dome	P2N1	1	19	0.468	0.059	0.750	fair	20	0.715	0.383	0.879	good	19	0.286	−0.157	0.642	poor
Dome	P2N1	1_2	20	0.603	0.242	0.820	good	20	0.583	0.209	0.810	fair	20	0.340	−0.093	0.670	poor
Dome	P2N1	1_5	20	0.674	0.345	0.856	good	20	0.522	0.135	0.775	fair	20	0.301	−0.134	0.645	poor
Trigone	P2N1	1	20	0.716	0.302	0.888	good	18	0.474	0.006	0.767	fair	16	0.616	0.201	0.845	good
Trigone	P2N1	1_2	20	0.882	0.603	0.958	excellent	20	0.558	0.153	0.799	fair	17	0.526	0.105	0.794	fair
Trigone	P2N1	1_5	20	0.866	0.697	0.944	excellent	20	0.618	0.245	0.830	good	20	0.583	0.216	0.809	fair
			**3.1** **Hz**														
Pudendus	P40	1_5	39	0.826	0.692	0.904	excellent										
Pudendus	N50	1_5	39	0.916	0.846	0.955	excellent										
Pudendus	P65	1_5	39	0.869	0.764	0.929	excellent										
Pudendus	N85	1_5	39	0.890	0.800	0.941	excellent										
Pudendus	P40N50	1_5	39	0.278	−0.042	0.545	poor										
Pudendus	P65N85	1_5	39	0.556	0.294	0.740	fair										

Intraclass correlation coefficient (ICC) values with confidence intervals for measures of P1, N1, P2 latency and P1N1 and P2N1 peak-to-peak amplitudes for the locations bladder dome (BD) and trigone (TG). In addition, ICC values are indicated for pudendal SSEP latencies (P40, N50, P65, N85) and peak-to-peak amplitudes (40N50, P65N85).

## Discussion

In this cohort of young healthy subjects we could successfully record cortical SEPs during bladder stimulation with three different frequencies (0.5 Hz, 1.1 Hz, 1.6 Hz – 1 ms pulse width) and evaluate the impact of several measurement settings, i.e. frequency, number of stimuli and data acquisition, on SEP outcome. Bladder SEPs with three clearly identifiable peaks, including a prominent negative deflection (N1) between two less positive deflections (P1 and P2), were observed with a 100% RR during stimulation with all applied stimulation frequencies across 500 stimuli. For both locations, significantly greater amplitudes were measured during stimulation with 0.5 Hz compared to 1.1 Hz and 1.6 Hz. This indicates that the choice of the stimulation frequency is crucial. This significant difference in amplitude values was shown based on the marker positions and on t-curves, respectively, as well as by SNR that include all of the measurements and information about the whole length of the segment. Lower stimulation frequencies might lead to larger amplitudes because of a better susceptibility of the slow fibers in the bladder to these frequencies due to longer refractory periods. N1 latency across 500 stimuli was found to be longer (15–25 ms) than in a few previous studies with healthy subjects^12,24,25^, but comparable to a study measuring SEPs in healthy young men^11^. Most of the previous studies performed the stimulation with 0.5–3 Hz. Nevertheless, the heterogeneous study populations and inconsistencies of measurement settings are likely the main reasons that hampered a meaningful frequency comparison. For the present study we decided to keep the pulse width constant at 1 ms for a proper comparison of the frequencies. Based on the knowledge of bladder fiber constitution and results from previous studies^8,11^, stimulation frequency 0.5 Hz and two frequencies below 3 Hz were selected.

An electrical phenomenon clearly visible in the SEP curves measured during stimulation with slow frequencies such as 0.5 Hz (Fig. 1) is a negative shift in the electroencephalography baseline. This observation is called contingent negative variation and can be attributed to attention and arousal function^26^.

Depending on the stimulation frequency, the application of 500 stimuli led to quite different stimulation durations (16.7 min for 0.5 Hz, 7.6 min for 1.1 Hz and 5.2 min for 1.6 Hz). Nevertheless, the comparison of the SEP curves averaged over a constant stimulation duration of 300 seconds showed similar results for the frequency comparison (Fig. 2). Consequently, we can exclude that the SEP differences observed between the stimulation frequencies (Fig. 1) resulted from varying duration.

Our data showed a gradual decrease in amplitudes, RR and SNR across runs (Figs. 3 and 4). Nevertheless, RR can be maximized by combination of at least two runs compared to the individual runs for all frequencies and the two bladder locations. The results suggest that the total number of stimuli or runs, respectively, can be reduced from 500 stimuli (5 runs) to 200 stimuli (2 runs) in order to achieve reliable bladder SEPs and at the same time minimize acquisition time. Until now it was unclear, how many stimuli are needed to get reliable bladder SEPs. Various numbers of applied stimuli (up to 1000 stimuli) and runs were reported previously for bladder SEP measurements^8,10,11^.

We assume that habituation or rapidly changing bladder volumes may lead to dislocation of the stimulation catheter or afferent inhibition, causing a decrease in amplitudes. The size of the SNR is important since bladder SEPs with bigger SNR are better detectable and thereby marker setting of P1, N1, P2 is easier. Although we are not bothered by increasing bladder volume over time during pudendal nerve stimulation, a significant, albeit smaller decrease in amplitudes was found across runs. This might be due to habituation even if the stimulation duration was much shorter.

Investigating SEP latencies and amplitudes, minimal differences between visits were found. Considering inter-individual variance and pudendal SSEPs these differences are negligible.

ICC analysis revealed good to excellent reliability for latencies of both bladder locations and for all three stimulation frequencies except for BD when stimulating with 1.6 Hz (Table 3). For the location BD, stimulation with 1.6 Hz showed the lowest ICC values, while the values were quite comparable for 0.5 Hz and 1.1 Hz. For the location TG, stimulation with 0.5 Hz showed better reliability compared to 1.1 Hz and 1.6 Hz for latency and amplitude values. Reliability is slightly better for the location TG compared to BD. Although the positioning of the electrodes is more difficult in the bladder compared to the skin, the ICC values of pudendal SEP latencies were comparable to N1 and P2 reliability of the lower stimulation frequencies for both bladder locations. In line with a previous study, reliability was higher for the latencies compared to the peak-to-peak amplitudes with better ICC for N1 latencies compared to P1 and P2^8^. The amplitudes are known to be more prone to changes compared to latencies since changing electrode impedances or varying relaxation degree of the participants can additionally influence it^27^. Compared to previous studies, reliability across visits was comparable or higher for all bladder SEP latencies and peak-to-peak amplitudes^8,28^. In addition to having a constant starting bladder volume of 60 mL, we compared the radiographs to be sure to place the catheter exactly at the same position at the two visits. Another important modification was to focus on setting the markers on the same SEP components across visits (see Fig. 3 in Knüpfer *et al*.^11^).

Previously, different low-pass filters were used for the preprocessing of bladder SEPs^8,11^. Our bladder SEP data do not confirm results from other fields reporting smaller amplitudes and longer latencies by decreasing low-pass filter^29–31^. However, the lack of difference in our study could be explained by the rather small difference between the two filters or to a certain part by the use of zero-phase shift filters in Vision Analyzer. Our choice of the 70 Hz low-pass filter rather affects high frequency components of the signal, which can explain the slightly smaller peak-to-peak amplitudes with the 70 Hz filter for the pudendal SEPs.

With respect to bladder SEP latencies and amplitudes, RR, reliability and SNR, our data suggest a stimulation frequency of 0.5 Hz to be the preferred technique for stimulation of the afferents of the BD and TG. These results were supported by analyses of the manually set markers and of the whole curve shape (t-curves). For the decision of the minimally required number of stimuli we have to consider the strong habituation effect and that this measurement should be applicable in patients where it will likely be more difficult to record reproducible SEPs. Although we observed the largest amplitudes and highest SNR during the first run, we may have a better RR when more stimuli are averaged so that according to the present results we would suggest a minimum of 2 runs of 100 stimuli. This is supported by the fact that a 100% RR was reached after 2 runs of stimulating with 0.5 Hz in both bladder locations, while these data showed good to excellent reliability across visits for the SEP latencies. N1 latency seems to be the robust marker with excellent reliability.

Our study provides a systematic bladder SEP evaluation in both gender groups while comparing different stimulation frequencies at two locations of the bladder. This data from healthy subjects was analyzed based on manual marker positions and on the whole SEP curve by means of standardized statistical testing (t-curves). It provides additional information on variability and potential confounding factors. The measurement of cortical SEPs elicited by bladder electrical stimulation may have the potential to serve as a neurophysiological biomarker for afferent nerve fiber function in patients with LUT symptoms such as OAB. By analysing latencies and peak-to-peak amplitudes of the SEP components one can obtain information on nerve fiber integrity and conduction velocity^7^. The application of bladder SEPs might be a useful amendment to findings from complementary investigations (i.e. history, neurological examination, urodynamic examination)^32^ as well as a surrogate marker and outcome measure for established and approved therapies targeting afferent bladder pathways. However, one should be aware of the rather high between-subject variability of bladder SEPs compared to the low variability within-subjects. Further studies testing this optimized setup in different neurological patient groups and age groups are needed and will help to define the clinical application field of this assessment tool. Additionally, consecutive investigations have to show if our bladder SEP findings hold true also for urethral stimulation sites, considering their distinct variations in afferent innervation and gender specific anatomy.

## Limitations

Currently, marker setting does not work automatically and consequently has to be performed manually, which is a subjective and very time-consuming task. Furthermore, the fact that different subjects were included per location complicates the direct comparison of BD and TG. Nevertheless, doing too many subsequent measurements in the bladder could influence the results due to irritations of the mucosa as well as attentional changes and decrease of compliance of the volunteers. Correspondingly, the observed decrease in amplitudes and RR with increasing number of stimuli (runs) may indicate habituation, which may be attributed to changes in attention. This may be avoided by introduction of a short random delay between single stimuli. This would have to be tested in further studies.

## Conclusion

The results of the present randomized study indicate that cortical potentials can be recorded in young healthy volunteers with high reliability and low repetition rates when stimulating the bladder with low frequencies. This potentially makes this investigation a clinically useful test for the investigation of bladder afferents. Our data clearly show that the choice of the stimulation parameters is very relevant for implementation of bladder SEPs into daily clinical practice. Based on the current results, we would recommend a stimulation frequency 0.5 Hz, because of best reproducibility, largest amplitudes and best SNR. N1 latency seems to be the most robust and reliable bladder SEP marker. The number of electrical stimuli can be reduced to 200 (2 runs of 100 stimuli) to achieve robust responses in reasonable acquisition time (400 seconds). This may constitute a good compromise between the duration of a stimulation cycle and peak-to-peak amplitudes of the SEP.

## Supplementary information

Supplementary information

## Data Availability

All data generated or analyzed in the framework of this manuscript are included in the published article.
