# Supplementary material for: Optimized Measurement Parameters of Sensory Evoked Cortical Potentials to Assess Human Bladder Afferents - A Randomized Study
Source: Sci Rep. 2019 Dec 20;9:19478. doi: 10.1038/s41598-019-54614-z (PMC6925113; doi:10.1038/s41598-019-54614-z)
Supplement: Supplementary file 1 — Supplementary information [file 41598_2019_54614_MOESM1_ESM.docx]

**SUPPLEMENTARY INFORMATION**

Optimized measurement parameters of sensory evoked cortical potentials to assess human bladder afferents - a randomized study

Stéphanie van der Lely^1,a^, Martina D. Liechti^1,a^, Melanie R. Schmidhalter^1^, Martin Schubert^2^, Lucas M. Bachmann^3^, Thomas M. Kessler^1^, Ulrich Mehnert^1*^

^1^ Department of Neuro-Urology, Balgrist University Hospital, University of Zürich, Zürich, Switzerland

^2^ Neurophysiology, Spinal Cord Injury Center, Balgrist University Hospital, University of Zürich, Zürich, Switzerland

^3^ Medignition Inc., Research Consultants, Zürich, Switzerland.

^a^ These authors shared the first authorship.

**FIGURES**

**
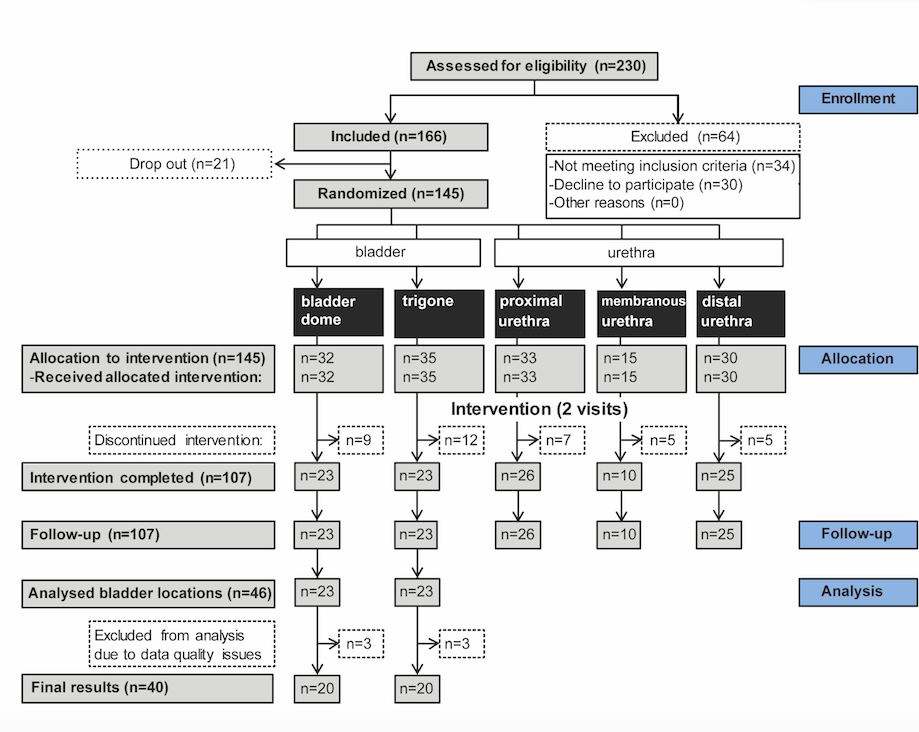
**

**Figure S1: Consort diagram for flow of participants through the study.** Reasons for discontinued intervention were the following: no participation in visit 2 (n=18), catheter could not be placed (n=14), uncomfrotable feeling caused by catheter/stimulation (n=5), poor health condition (n=1).

**
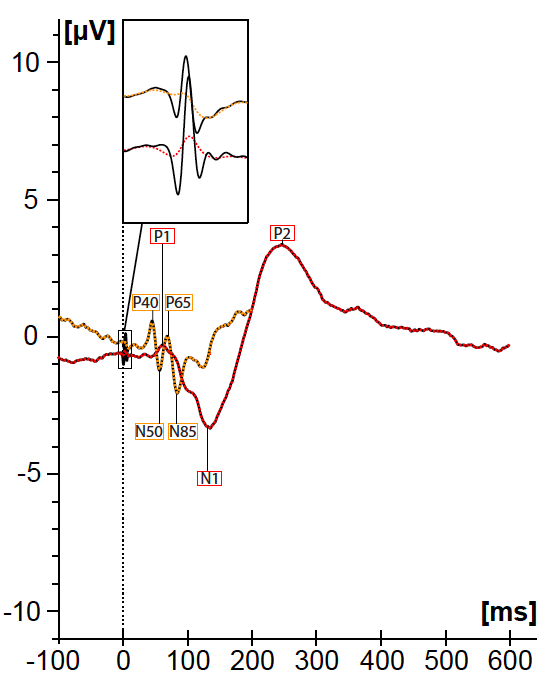
**

**Figure S2: Overlay of 70Hz and 200Hz low-pass filtered bladder (n=5) and pudendal SEPs (n=9), respectively.** Averaged bladder SEPs (BD and TG) processed with 70Hz low-pass filter are shown in red, pudendal SEPs in orange. Data processed with 200Hz are shown in black. Zoomed-in sections are shown for the stimulus artifact.

**TABLES**

**Table S1: Stimulation parameters and bladder volume stratified for location, gender and frequency.**

|  |  |  |  |  |  | | **Group effects** | | | | | |
| --- | --- | --- | --- | --- | --- | --- | --- | --- | --- | --- | --- | --- |
|  |  | **Women Dome** | **Men Dome** | **Women Trig** | **Men Trigone** | | **Gender - Dome** | | **Gender - Trigone** | | **Location** | |
|  | ***Frequency*** | **(n=10)** | **(n=10)** | **(n=10)** | **(n=10)** | | **p-value** | | **p-value** | | **p-value** | |
| **CPT [mA]** | *0.5Hz* | 5.5 (2.8-10.2) | 6.8 (2.9-17.1) | 4.1 (1.4-17.9) | 3.5 (1.4-11.2) | 0.160 | |  | 0.695 |  | <0.001 | * |
|  | *1.1Hz* | 4.8 (1.6-9.8) | 7.2 (2.8-17.1) | 3.9 (2.2-29.7) | 3.8 (1.6-8.5) | 0.015 | | * | 0.860 |  | 0.001 | * |
|  | *1.6Hz* | 5.1 (1.8-12.0) | 7.5 (1-15.8) | 4.1 (1.4-9.5) | 3.9 (1.9-14.4) | 0.088 | |  | 1.000 |  | <0.001 | * |
|  | *Pudendus - 3.1Hz* | 2.7 (1.4- 4.9) | 3.0 (1.5-5.8) | 2.6 (1.2-3.8) | 2.5 (1.1-5.3) | 0.871 | |  | 0.892 |  | 0.262 |  |
| **Absolute stimulation intensity [mA]** | *0.5Hz* | 19.0 (7.1-41.6) | 28.4 (15.2-58.4) | 18.8 (7.3-51.0) | 15.3 (5.7-52.2) | 0.017 | | * | 0.636 |  | 0.004 | * |
|  | *1.1Hz* | 14.4 (5.4-31.8) | 23.8 (9.2-47.8) | 14.1 (8.4-56.8) | 12.6 (6.5-37.8) | <0.001 | | * | 0.323 |  | 0.031 | * |
|  | *1.6Hz* | 15.3 (6.2-22.6) | 23.7 (9.5-39.2) | 12.0 (9.5-31.6) | 11.5 (5.8-36.4) | 0.002 | | * | 0.365 |  | 0.008 | * |
|  | *Pudendus - 3.1Hz* | 9.0 (4.1-20.8) | 16.5 (5-33) | 8.6 (4.5-13.1) | 15.3 (6.8-32.6) | 0.002 | | * | <0.001 | * | 0.400 |  |
| **Relative stimulation intensity** | *0.5Hz* | 3.1 (2.2 -7.3) | 3.4 (2.2-8.6) | 4.3 (2.5 -8.7) | 5.4 (1.4-15.3) | 0.183 | |  | 0.301 |  | 0.039 | * |
|  | *1.1Hz* | 3.3 (2.1-5.1) | 3.6 (2.1-7.1) | 3.8 (1.9-6.5) | 3.7 (1.7-9.4) | 0.482 | |  | 0.925 |  | 0.127 |  |
|  | *1.6Hz* | 2.6 (1.7-5.1) | 3.2 (1.7-9.6) | 3.3 (2.3-7.0) | 3.9 (1.6-8.2) | 0.495 | |  | 0.862 |  | 0.090 |  |
|  | *Pudendus - 3.1Hz* | 3.2 (1.5-7.3) | 5.9 (2.9-13.1) | 3.2 (2.2 -5.7) | 6.3 (3.2 -13.7) | <0.001 | | * | <0.001 | * | 0.966 |  |
| **Produced volume [mL]** | *0.5Hz* | 165 (30-280) | 100 (10-270) | 130 (10-300) | 115 (40-670) | 0.010 | | * | 0.914 |  | 0.904 |  |
|  | *1.1Hz* | 135 (40-280) | 60 (0-150) | 125 (18-250) | 80 (20-240) | <0.001 | | * | 0.455 |  | 0.710 |  |
|  | *1.6Hz* | 90 (30-240) | 50 (0-140) | 85 (8-180) | 50 (10-340) | 0.040 | | * | 0.712 |  | 0.877 |  |

Values are reported with median and range. Significant differences are marked: *p<0.05.

**Table S2: Results of the LMM showing the influence of different variables on P1N1 amplitude.**

|  | **Dome, 1.visit** | | | | | **Dome, 2. visit** | | | | | **Trigone, 1. visit** | | | | | **Trigone, 2. visit** | | | | |
| --- | --- | --- | --- | --- | --- | --- | --- | --- | --- | --- | --- | --- | --- | --- | --- | --- | --- | --- | --- | --- |
|  | **Estimate**  **[ms]** | **Pr(>\|t\|)** | | **CI**  **lower** | **CI**  **Upper** | **Estimate**  **[ms]** | **Pr(>\|t\|)** | | **CI**  **lower** | **CI**  **Upper** | **Estimate**  **[ms]** | **Pr(>\|t\|)** | | **CI**  **lower** | **CI**  **Upper** | **Estimate**  **[ms]** | **Pr(>\|t\|)** | | **CI**  **lower** | **CI**  **Upper** |
| (Intercept) | -1.82 | 0.87 |  | -23.89 | 20.25 | 33.51 | 0.10 |  | -6.67 | 73.70 | 0.35 | 0.96 |  | -14.20 | 14.89 | 14.42 | 0.15 |  | -5.94 | 34.78 |
| Freq. 1.1Hz | -2.58 | 0.00 | * | -3.93 | -1.23 | -2.20 | 0.00 | * | -3.65 | -0.76 | -1.36 | 0.00 | * | -2.05 | -0.67 | -1.36 | 0.00 | * | -2.17 | -0.55 |
| Freq. 1.6Hz | -3.93 | 0.00 | * | -5.48 | -2.39 | -3.50 | 0.00 | * | -5.25 | -1.74 | -1.74 | 0.01 | * | -2.94 | -0.54 | -1.83 | 0.00 | * | -2.94 | -0.71 |
|  |  |  |  |  |  |  |  |  |  |  |  |  |  |  |  |  |  |  |  |  |
| Run2 | -1.15 | 0.00 | * | -1.74 | -0.57 | -0.75 | 0.01 | * | -1.32 | -0.18 | 0.18 | 0.63 |  | -0.60 | 0.97 | -0.06 | 0.80 |  | -0.59 | 0.46 |
| Run3 | -1.62 | 0.00 | * | -2.41 | -0.82 | -1.46 | 0.00 | * | -2.22 | -0.71 | -0.10 | 0.81 |  | -0.92 | 0.73 | -0.59 | 0.02 |  | -1.09 | -0.10 |
| Run4 | -1.86 | 0.00 | * | -2.56 | -1.16 | -1.90 | 0.00 | * | -2.81 | -0.99 | -0.31 | 0.45 |  | -1.13 | 0.52 | -0.66 | 0.01 | * | -1.15 | -0.16 |
| Run5 | -2.22 | 0.00 | * | -3.23 | -1.21 | -2.27 | 0.00 | * | -3.20 | -1.35 | -0.33 | 0.40 |  | -1.14 | 0.47 | -0.77 | 0.01 | * | -1.36 | -0.18 |
| Run1_2 | -0.77 | 0.00 | * | -1.14 | -0.40 | -0.47 | 0.00 | * | -0.74 | -0.19 | -0.10 | 0.61 |  | -0.48 | 0.29 | -0.18 | 0.12 |  | -0.41 | 0.05 |
| Run1_3 | -1.24 | 0.00 | * | -1.76 | -0.72 | -0.93 | 0.00 | * | -1.39 | -0.48 | -0.33 | 0.19 |  | -0.83 | 0.18 | -0.44 | 0.01 | * | -0.75 | -0.14 |
| Run1_4 | -1.57 | 0.00 | * | -2.20 | -0.94 | -1.35 | 0.00 | * | -1.96 | -0.74 | -0.49 | 0.09 |  | -1.05 | 0.08 | -0.62 | 0.00 | * | -0.96 | -0.28 |
| Run1_5 | -1.85 | 0.00 | * | -2.55 | -1.16 | -1.75 | 0.00 | * | -2.45 | -1.06 | -0.59 | 0.05 |  | -1.18 | -0.01 | -0.75 | 0.00 | * | -1.12 | -0.38 |
|  |  |  |  |  |  |  |  |  |  |  |  |  |  |  |  |  |  |  |  |  |
| Gender-male | -1.96 | 0.06 |  | -4.02 | 0.11 | -0.69 | 0.58 |  | -3.28 | 1.89 | 0.82 | 0.34 |  | -0.94 | 2.57 | 0.76 | 0.38 |  | -1.03 | 2.56 |
| Age | -0.13 | 0.30 |  | -0.39 | 0.13 | -0.21 | 0.16 |  | -0.51 | 0.09 | -0.08 | 0.36 |  | -0.24 | 0.09 | -0.08 | 0.27 |  | -0.22 | 0.06 |
| Body weight | 0.07 | 0.29 |  | -0.06 | 0.19 | 0.17 | 0.04 | * | 0.01 | 0.33 | 0.03 | 0.64 |  | -0.10 | 0.17 | 0.08 | 0.26 |  | -0.07 | 0.23 |
| Volume | 0.00 | 0.74 |  | -0.02 | 0.01 | -0.01 | 0.26 |  | -0.03 | 0.01 | 0.00 | 0.69 |  | -0.01 | 0.02 | 0.00 | 0.69 |  | -0.01 | 0.00 |
| Body height | 0.07 | 0.36 |  | -0.08 | 0.22 | -0.17 | 0.19 |  | -0.43 | 0.09 | 0.02 | 0.75 |  | -0.10 | 0.14 | -0.07 | 0.38 |  | -0.24 | 0.09 |
| Intensity | -0.06 | 0.20 |  | -0.17 | 0.04 | -0.05 | 0.29 |  | -0.15 | 0.05 | 0.03 | 0.47 |  | -0.05 | 0.11 | -0.04 | 0.17 |  | -0.10 | 0.02 |

adjusted R^2^ = 0.370 / 0.277 / 0.247 / 0.184

Significant differences are marked: *p<0.05. Volume=Produced volume in mL, Intensity= absolute stimulation intensity in mA.

**Table S3: Results of the LMM showing the influence of different variables on P1 latency.**

|  | **Dome, 1.visit** | | | | | **Dome, 2. visit** | | | | | **Trigone, 1. visit** | | | | | **Trigone, 2. visit** | | | | |
| --- | --- | --- | --- | --- | --- | --- | --- | --- | --- | --- | --- | --- | --- | --- | --- | --- | --- | --- | --- | --- |
|  | **Estimate**  **[ms]** | **Pr(>\|t\|)** | | **CI**  **lower** | **CI**  **Upper** | **Estimate**  **[ms]** | **Pr(>\|t\|)** | | **CI**  **lower** | **CI**  **Upper** | **Estimate**  **[ms]** | **Pr(>\|t\|)** | | **CI**  **lower** | **CI**  **Upper** | **Estimate**  **[ms]** | **Pr(>\|t\|)** | | **CI**  **lower** | **CI**  **Upper** |
| (Intercept) | 36.63 | 0.30 |  | -34.72 | 107.98 | 36.31 | 0.29 |  | -33.49 | 106.11 | 36.59 | 0.02 |  | 7.30 | 65.87 | 50.60 | 0.05 |  | -0.72 | 101.93 |
| Freq. 1.1Hz | 2.72 | 0.07 |  | -0.21 | 5.64 | 1.26 | 0.53 |  | -2.85 | 5.37 | -0.08 | 0.95 |  | -2.54 | 2.39 | -0.12 | 0.92 |  | -2.67 | 2.44 |
| Freq. 1.6Hz | 8.56 | 0.00 | * | 3.80 | 13.32 | 2.45 | 0.18 |  | -1.28 | 6.19 | 2.58 | 0.19 |  | -1.39 | 6.55 | 0.80 | 0.55 |  | -1.92 | 3.51 |
|  |  |  |  |  |  |  |  |  |  |  |  |  |  |  |  |  |  |  |  |  |
| Run2 | 0.71 | 0.21 |  | -0.43 | 1.85 | 0.55 | 0.31 |  | -0.54 | 1.64 | -0.68 | 0.39 |  | -2.30 | 0.94 | 1.22 | 0.08 |  | -0.14 | 2.58 |
| Run3 | 0.64 | 0.40 |  | -0.93 | 2.22 | 1.50 | 0.00 | * | 0.58 | 2.42 | -1.01 | 0.06 |  | -2.05 | 0.04 | 0.41 | 0.64 |  | -1.39 | 2.22 |
| Run4 | 1.57 | 0.07 |  | -0.16 | 3.30 | 0.46 | 0.47 |  | -0.86 | 1.79 | -0.38 | 0.61 |  | -1.93 | 1.17 | 0.97 | 0.39 |  | -1.31 | 3.24 |
| Run5 | 1.88 | 0.01 | * | 0.44 | 3.32 | 1.87 | 0.03 | * | 0.25 | 3.49 | -1.38 | 0.06 |  | -2.84 | 0.08 | -0.70 | 0.30 |  | -2.07 | 0.67 |
| Run1_2 | 0.42 | 0.28 |  | -0.37 | 1.21 | 0.41 | 0.18 |  | -0.20 | 1.02 | -0.20 | 0.74 |  | -1.48 | 1.07 | 0.63 | 0.12 |  | -0.18 | 1.44 |
| Run1_3 | 0.39 | 0.36 |  | -0.47 | 1.25 | 0.69 | 0.03 | * | 0.08 | 1.29 | -0.03 | 0.96 |  | -0.94 | 0.89 | 0.69 | 0.04 | * | 0.04 | 1.34 |
| Run1_4 | 0.57 | 0.20 |  | -0.34 | 1.47 | 0.76 | 0.04 | * | 0.05 | 1.47 | -0.08 | 0.88 |  | -1.11 | 0.96 | 0.67 | 0.11 |  | -0.17 | 1.52 |
| Run1_5 | 0.79 | 0.09 |  | -0.15 | 1.72 | 0.95 | 0.01 | * | 0.32 | 1.59 | 0.28 | 0.60 |  | -0.81 | 1.36 | 0.43 | 0.37 |  | -0.55 | 1.41 |
|  |  |  |  |  |  |  |  |  |  |  |  |  |  |  |  |  |  |  |  |  |
| Gender-male | 1.13 | 0.80 |  | -8.04 | 10.31 | -3.18 | 0.49 |  | -12.59 | 6.23 | 3.22 | 0.42 |  | -4.92 | 11.36 | 2.93 | 0.50 |  | -6.07 | 11.94 |
| Age | 0.45 | 0.19 |  | -0.24 | 1.13 | 0.92 | 0.02 | * | 0.17 | 1.66 | -0.31 | 0.34 |  | -0.98 | 0.36 | -0.41 | 0.40 |  | -1.41 | 0.59 |
| Body weight | -0.42 | 0.10 |  | -0.92 | 0.09 | -0.15 | 0.53 |  | -0.64 | 0.34 | -0.41 | 0.11 |  | -0.92 | 0.10 | -0.20 | 0.38 |  | -0.68 | 0.27 |
| Volume | 0.03 | 0.11 |  | -0.01 | 0.06 | -0.01 | 0.72 |  | -0.05 | 0.03 | 0.01 | 0.51 |  | -0.03 | 0.06 | -0.01 | 0.37 |  | -0.03 | 0.01 |
| Body height | 0.23 | 0.31 |  | -0.24 | 0.70 | 0.11 | 0.61 |  | -0.33 | 0.55 | 0.31 | 0.02 | * | 0.05 | 0.56 | 0.19 | 0.29 |  | -0.17 | 0.55 |
| Intensity | -0.07 | 0.67 |  | -0.38 | 0.25 | -0.06 | 0.70 |  | -0.41 | 0.28 | 0.13 | 0.37 |  | -0.17 | 0.42 | -0.03 | 0.73 |  | -0.22 | 0.16 |

adjusted R^2^ = 0.283 / 0.270 / 0.150 / 0.112

Significant differences are marked: *p<0.05. Volume=Produced volume in mL, Intensity= absolute stimulation intensity in mA.

**Table S4: Results of the LMM showing the influence of different variables on P2 latency.**

|  | **Dome, 1.visit** | | | | | **Dome, 2. visit** | | | | | **Trigone, 1. visit** | | | | | **Trigone, 2. visit** | | | | |
| --- | --- | --- | --- | --- | --- | --- | --- | --- | --- | --- | --- | --- | --- | --- | --- | --- | --- | --- | --- | --- |
|  | **Estimate**  **[ms]** | **Pr(>\|t\|)** | | **CI**  **lower** | **CI**  **Upper** | **Estimate**  **[ms]** | **Pr(>\|t\|)** | | **CI**  **lower** | **CI**  **Upper** | **Estimate**  **[ms]** | **Pr(>\|t\|)** | | **CI**  **lower** | **CI**  **Upper** | **Estimate**  **[ms]** | **Pr(>\|t\|)** | | **CI**  **lower** | **CI**  **Upper** |
| (Intercept) | 6.57 | 0.95 |  | -197.89 | 211.03 | -8.21 | 0.95 |  | -287.22 | 270.80 | 206.15 | 0.00 |  | 102.21 | 310.09 | 125.85 | 0.09 |  | -19.12 | 270.81 |
| Freq. 1.1Hz | 10.98 | 0.01 | * | 2.96 | 19.01 | 5.76 | 0.21 |  | -3.50 | 15.02 | 2.69 | 0.42 |  | -4.07 | 9.44 | 2.32 | 0.54 |  | -5.54 | 10.18 |
| Freq. 1.6Hz | 13.57 | 0.01 | * | 3.02 | 24.12 | 1.94 | 0.69 |  | -8.22 | 12.10 | 1.40 | 0.80 |  | -9.98 | 12.78 | -1.45 | 0.70 |  | -9.14 | 6.23 |
|  |  |  |  |  |  |  |  |  |  |  |  |  |  |  |  |  |  |  |  |  |
| Run2 | 0.54 | 0.52 |  | -1.19 | 2.27 | 0.04 | 0.97 |  | -2.49 | 2.57 | -4.14 | 0.04 | * | -8.04 | -0.23 | 0.81 | 0.59 |  | -2.25 | 3.87 |
| Run3 | 1.78 | 0.16 |  | -0.74 | 4.29 | 2.91 | 0.14 |  | -1.08 | 6.90 | -0.37 | 0.82 |  | -3.74 | 3.01 | -1.11 | 0.50 |  | -4.50 | 2.28 |
| Run4 | 2.19 | 0.17 |  | -1.01 | 5.38 | 2.69 | 0.19 |  | -1.41 | 6.79 | -2.10 | 0.41 |  | -7.34 | 3.15 | -2.10 | 0.28 |  | -6.08 | 1.88 |
| Run5 | -0.17 | 0.91 |  | -3.31 | 2.98 | 3.14 | 0.17 |  | -1.50 | 7.79 | -3.99 | 0.07 |  | -8.29 | 0.31 | -0.35 | 0.83 |  | -3.74 | 3.04 |
| Run1_2 | 0.51 | 0.53 |  | -1.15 | 2.16 | 1.00 | 0.18 |  | -0.52 | 2.52 | 0.34 | 0.75 |  | -1.81 | 2.48 | 0.74 | 0.35 |  | -0.87 | 2.35 |
| Run1_3 | 1.05 | 0.18 |  | -0.53 | 2.64 | 2.01 | 0.03 | * | 0.22 | 3.80 | 1.67 | 0.14 |  | -0.61 | 3.95 | 2.13 | 0.02 | * | 0.40 | 3.85 |
| Run1_4 | 1.26 | 0.13 |  | -0.39 | 2.92 | 2.85 | 0.02 | * | 0.48 | 5.23 | 2.27 | 0.07 |  | -0.16 | 4.71 | 2.32 | 0.01 | * | 0.57 | 4.07 |
| Run1_5 | 1.69 | 0.07 |  | -0.13 | 3.51 | 3.20 | 0.02 | * | 0.60 | 5.81 | 2.12 | 0.06 |  | -0.10 | 4.35 | 2.43 | 0.02 | * | 0.39 | 4.47 |
|  |  |  |  |  |  |  |  |  |  |  |  |  |  |  |  |  |  |  |  |  |
| Gender-male | -3.88 | 0.74 |  | -28.39 | 20.63 | -1.53 | 0.90 |  | -26.27 | 23.21 | 4.43 | 0.53 |  | -10.11 | 18.97 | -2.29 | 0.73 |  | -15.89 | 11.31 |
| Age | 2.96 | 0.03 | * | 0.30 | 5.63 | 3.29 | 0.06 |  | -0.09 | 6.67 | -0.31 | 0.73 |  | -2.14 | 1.52 | -0.56 | 0.53 |  | -2.42 | 1.29 |
| Body weight | -0.76 | 0.34 |  | -2.36 | 0.85 | -0.37 | 0.60 |  | -1.84 | 1.10 | -0.96 | 0.10 |  | -2.13 | 0.21 | -1.25 | 0.05 |  | -2.49 | -0.01 |
| Volume | 0.04 | 0.42 |  | -0.07 | 0.15 | 0.03 | 0.48 |  | -0.06 | 0.13 | -0.01 | 0.90 |  | -0.11 | 0.09 | -0.04 | 0.25 |  | -0.10 | 0.03 |
| Body height | 1.19 | 0.13 |  | -0.36 | 2.74 | 1.15 | 0.26 |  | -0.94 | 3.23 | 0.68 | 0.13 |  | -0.21 | 1.56 | 1.35 | 0.04 | * | 0.05 | 2.64 |
| Intensity | 0.75 | 0.04 | * | 0.02 | 1.47 | 0.06 | 0.89 |  | -0.78 | 0.90 | 0.22 | 0.57 |  | -0.56 | 0.99 | -0.03 | 0.92 |  | -0.56 | 0.51 |

adjusted R^2^ = 0.279 / 0.266 / 0.097 / 0.225

Significant differences are marked: *p<0.05. Volume=Produced volume in mL, Intensity= absolute stimulation intensity in mA.

**Table S5: Results of the LMM showing the influence of different variables on P40N50 amplitude.**

|  | **Pudendus, 1.visit** | | | | | **Pudendus, 2. visit** | | | | |
| --- | --- | --- | --- | --- | --- | --- | --- | --- | --- | --- |
|  | **Estimate**  **[μV]** | **Pr(>\|t\|)** | | **CI**  **lower** | **CI**  **Upper** | **Estimate**  **[μV]** | **Pr(>\|t\|)** | | **CI**  **lower** | **CI**  **Upper** |
| (Intercept) | 5.58 | 0.23 |  | -3.67 | 14.83 | 7.63 | 0.01 |  | 1.86 | 13.39 |
| Run2 | 0.05 | 0.69 |  | -0.20 | 0.30 | 0.01 | 0.93 |  | -0.24 | 0.26 |
| Run3 | 0.00 | 0.98 |  | -0.26 | 0.26 | -0.16 | 0.29 |  | -0.45 | 0.14 |
| Run4 | -0.14 | 0.29 |  | -0.41 | 0.13 | -0.26 | 0.09 |  | -0.57 | 0.04 |
| Run5 | -0.27 | 0.03 | * | -0.50 | -0.03 | -0.23 | 0.13 |  | -0.53 | 0.07 |
| Run1_2 | -0.10 | 0.15 |  | -0.24 | 0.04 | -0.08 | 0.29 |  | -0.24 | 0.07 |
| Run1_3 | -0.10 | 0.20 |  | -0.25 | 0.06 | -0.15 | 0.11 |  | -0.33 | 0.03 |
| Run1_4 | -0.13 | 0.14 |  | -0.30 | 0.05 | -0.20 | 0.05 |  | -0.41 | 0.00 |
| Run1_5 | -0.19 | 0.03 | * | -0.35 | -0.02 | -0.24 | 0.02 | * | -0.44 | -0.03 |
|  |  |  |  |  |  |  |  |  |  |  |
| Gender | -0.07 | 0.79 |  | -0.63 | 0.48 | 0.72 | 0.15 |  | -0.28 | 1.72 |
| Age | 0.00 | 0.90 |  | -0.07 | 0.08 | 0.00 | 0.96 |  | -0.10 | 0.09 |
| Body weight | 0.03 | 0.22 |  | -0.02 | 0.08 | 0.08 | 0.01 | * | 0.02 | 0.14 |
| Body height | -0.04 | 0.25 |  | -0.10 | 0.03 | -0.06 | 0.01 | * | -0.11 | -0.02 |
| Intensity | 0.04 | 0.16 |  | -0.01 | 0.09 | -0.03 | 0.39 |  | -0.10 | 0.04 |

adjusted R^2^ = 0.097 / 0.173

Significant differences are marked: *p<0.05. Intensity= absolute stimulation intensity in mA.

**Table S6: Results of the LMM showing the influence of different variables on P40 latency.**

|  | **Pudendus, 1.visit** | | | | | **Pudendus, 2. visit** | | | | |
| --- | --- | --- | --- | --- | --- | --- | --- | --- | --- | --- |
|  | **Estimate**  **[ms]** | **Pr(>\|t\|)** | | **CI**  **lower** | **CI**  **Upper** | **Estimate**  **[ms]** | **Pr(>\|t\|)** | | **CI**  **lower** | **CI**  **Upper** |
| (Intercept) | 33.10 | 0.01 |  | 8.89 | 57.31 | 30.03 | 0.01 |  | 6.33 | 53.73 |
| Run2 | 0.17 | 0.59 |  | -0.48 | 0.83 | 0.14 | 0.73 |  | -0.70 | 1.00 |
| Run3 | 0.39 | 0.23 |  | -0.27 | 1.05 | 0.02 | 0.94 |  | -0.58 | 0.63 |
| Run4 | 0.28 | 0.44 |  | -0.45 | 1.02 | 0.11 | 0.81 |  | -0.83 | 1.05 |
| Run5 | 0.25 | 0.45 |  | -0.42 | 0.92 | -0.55 | 0.40 |  | -1.87 | 0.76 |
| Run1_2 | 0.23 | 0.35 |  | -0.26 | 0.72 | 0.05 | 0.87 |  | -0.60 | 0.70 |
| Run1_3 | 0.35 | 0.14 |  | -0.12 | 0.82 | -0.05 | 0.88 |  | -0.73 | 0.63 |
| Run1_4 | 0.33 | 0.18 |  | -0.16 | 0.83 | 0.04 | 0.91 |  | -0.61 | 0.68 |
| Run1_5 | 0.27 | 0.22 |  | -0.17 | 0.71 | -0.14 | 0.69 |  | -0.87 | 0.58 |
|  |  |  |  |  |  |  |  |  |  |  |
| Gender | 3.86 | 0.00 | * | 1.84 | 5.88 | 3.57 | 0.00 | * | 1.42 | 5.71 |
| Age | 0.07 | 0.57 |  | -0.17 | 0.31 | 0.12 | 0.19 |  | -0.06 | 0.31 |
| Body weight | 0.02 | 0.77 |  | -0.13 | 0.17 | -0.02 | 0.76 |  | -0.18 | 0.14 |
| Body height | 0.04 | 0.62 |  | -0.12 | 0.20 | 0.06 | 0.43 |  | -0.10 | 0.22 |
| Intensity | -0.14 | 0.05 |  | -0.28 | 0.00 | -0.04 | 0.53 |  | -0.17 | 0.09 |

adjusted R^2^ = 0.290 / 0.278

Significant differences are marked: *p<0.05. Intensity= absolute stimulation intensity in mA.

**Table S7: Results of the LMM showing the influence of different variables on N50 latency.**

|  | **Pudendus, 1.visit** | | | | | **Pudendus, 2. visit** | | | | |
| --- | --- | --- | --- | --- | --- | --- | --- | --- | --- | --- |
|  | **Estimate**  **[ms]** | **Pr(>\|t\|)** | | **CI**  **lower** | **CI**  **Upper** | **Estimate**  **[ms]** | **Pr(>\|t\|)** | | **CI**  **lower** | **CI**  **Upper** |
| (Intercept) | 43.17 | 0.00 |  | 14.23 | 72.11 | 39.42 | 0.01 |  | 11.00 | 67.84 |
| Run2 | 0.35 | 0.27 |  | -0.29 | 0.99 | 0.31 | 0.24 |  | -0.22 | 0.84 |
| Run3 | 0.35 | 0.34 |  | -0.38 | 1.07 | 0.14 | 0.65 |  | -0.46 | 0.74 |
| Run4 | 0.54 | 0.12 |  | -0.15 | 1.23 | -0.32 | 0.22 |  | -0.84 | 0.20 |
| Run5 | 0.04 | 0.90 |  | -0.63 | 0.71 | 0.00 | 1.00 |  | -0.61 | 0.61 |
| Run1_2 | 0.28 | 0.21 |  | -0.17 | 0.73 | 0.11 | 0.55 |  | -0.24 | 0.45 |
| Run1_3 | 0.46 | 0.10 |  | -0.09 | 1.02 | 0.01 | 0.95 |  | -0.37 | 0.40 |
| Run1_4 | 0.49 | 0.08 |  | -0.07 | 1.04 | 0.05 | 0.80 |  | -0.34 | 0.44 |
| Run1_5 | 0.39 | 0.12 |  | -0.10 | 0.89 | -0.02 | 0.93 |  | -0.42 | 0.38 |
|  |  |  |  |  |  |  |  |  |  |  |
| Gender | 4.39 | 0.00 | * | 2.00 | 6.77 | 4.77 | 0.00 | * | 2.31 | 7.24 |
| Age | 0.21 | 0.09 |  | -0.04 | 0.46 | 0.20 | 0.04 | * | 0.01 | 0.40 |
| Body weight | -0.01 | 0.90 |  | -0.19 | 0.16 | -0.02 | 0.82 |  | -0.18 | 0.14 |
| Body height | 0.04 | 0.72 |  | -0.16 | 0.24 | 0.05 | 0.58 |  | -0.14 | 0.25 |
| Intensity | -0.06 | 0.46 |  | -0.21 | 0.10 | 0.02 | 0.81 |  | -0.16 | 0.20 |

adjusted R^2^ = 0.328 / 0.450

Significant differences are marked: *p<0.05. Intensity= absolute stimulation intensity in mA.
